# Transactions of the Medical Society of the County of Kings

**Published:** 1865-01

**Authors:** 


BUFFALO

### Medical and Surgical Journal.

VOL. IV. JANUARY, 1865. No. 6.

### *Transactions of the Medical Society of the County of Kings.*

REGULAR MEETING, NOVEMBER, 1862.

### *Fracture of the Skull with Depression—Removal of the Fragments.*

Dr. WILLIAM GILFILLAN reported the following case:

M. D. Cashow, aged 18, private in the 1st Connecticut Artillery, was wounded in the head June 27th at Savage's Station, Va. The injury was received in a charge of Stuart's cavalry. A horse struck him on the back of the head, and he was immediately stunned, and remained unconscious for some hours. No careful examination of the wound was made for some weeks. Water dressing was applied, and pus was freely secreted. In the beginning of September, while at Baltimore, he was discharged from the service of the United States. On the 26th of September, he was admitted to the Long Island College Hospital. He was then in the full possession of all his faculties—countenance was a little pale. Exactly at the occipital protuberance there was a wound of scalp, admitting the tip of the little finger. On introducing a probe, the bone was felt denuded and rough over a space of two inches or more. At one point the probe passed between the bone and the fragments and touched the duramater. The necrosed fragment extended more to the right than to the left of the middle line. The pus presented the peculiar odor of dead bone. The dead piece of bone could not be moved by the probe. After consultation, it was determined to reflect the scalp by a T shaped incision, and remove the dead pieces of bone, as from the length of time which had elapsed since the receipt of the injury separation must have taken place.

On the 28th, the patient being under the influence of chloroform, I cut down and removed two pieces of bone. Some traction was requisite to free them from the soft parts. When the bones were removed, the duramater was exposed to sight and touch for a space of fully three by two inches.

The flaps were brought in position and water dressing applied, and morphia, gr. $\frac{1}{8}$, administered. He felt great pain in the wound, and after some hours, vomited. Of the pieces of bone removed, the larger measured one and a half by two and a quarter inches; the smaller measured one-half by two inches. Considerable discharge of pus took place for some days afterwards. Two weeks after the operation an abscess formed below the occipital protuberance, and was opened, discharging a large amount of fetid pus. In three weeks it had quite healed, and on the 20th of October he was discharged cured. He immediately enlisted in the 18th Connecticut Volunteers, and expressed his determination to avenge the loss of his occipital bone.

There are several points of interest in this case:

I. The absence of symptoms of compression at any time, although the bone was depressed under the edge of the remaining sound piece of occipital bone.

II. The want of physiological symptoms. From its position exactly over the occipital protuberance, we should expect *a priori* some disturbance in the functions of the cerebellum; but his sexual propensities were unaffected, and his power of locomotion and muscular co-ordination unimpeded.

III. The loss of such a large portion of the vault of the cranium, fully three inches by at least two and a half inches, and the ready healing of the soft parts over it.

IV. The fact that only the external table is represented in these pieces removed, although the duramater was fully exposed. What became of the internal table? Was it dissolved in the pus, which was discharging freely for three months?

### *Tuberculous tumor of the brain.*

Dr. John Ball presented a tumor taken from the brain of a negro, who had been subject to frequent attacks of convulsions since the 21st July last—the time of his first having seen him.

Since that time he had been called upon twice only, to see him, at several weeks interval, and therefore had lost sight of him until three days ago he was called upon to give a certificate of death, the man having suddenly died. Coroner Norris was notified, and, assisted by Dr. Minor he made a post mortem examination five hours after death, when the specimen presented—*a tuberculous tumor*—connected with bony deposit was found attached to the duramater and falx-cerebri of the right hemisphere of the brain.

Dr. Minor considered the remarkable features of the case to be death several days subsequent to the last convulsion, with so large an extravasation of dark blood, and the deceptive appearance of the tumor, which was apparently fibrous until the microscope revealed it to be tuberculous. So far as could be ascertained there was no tuberculous diathesis.

Dr. Gilfillan cited a case that occurred in his practice sometime ago, where there was similar extravasation, he thought in consequence of the rupture of some of the large vessels, and perhaps the position of the head at the time of death. In still another case that he had examined, that died with hypertrophy of the heart, at least a quart of blood flowed from the opened skull.

Dr. Enos compared the tumor in Dr. Ball's case, to those sometimes found on the surface of the liver and in the knee joint. It was also remarkable for its situation in another respect—the deposit of bony matter *in the falx*—instead of the duramater where we should most look for it. The falx in the lower animals is frequently ossified, and this case suggests the query whether it is not more likely to be so in the negro than in the white man. Another peculiarity was, that the adventitious bone was devoid of haversian canals, but why, he could not understand.

Dr. Jones referred to a post mortem examination of a case of permature labour in a negro woman who died in a "fit", where he found the falx cerebri completely ossified. There were also several clots in the vicinity of the ossified falx, but no extravasation. This case was also remarkable from having *twenty-eight uterine tumors*, some fibrous and others calcareous.

---

### REGULAR MEETING, DECEMBER, 1862.

### *Resection of the Ankle — Recovery with three-fourths of an inch shortening.*

By Dr. John G. Johnson.

In this age of conservative surgery, when resections are so much in favor, when operations upon the largest articulations can be found in numbers in almost every periodical, those upon the ankle-joint seem strangely few. The hip, knee and elbow are the favorite articulations for resection, while at the ankle, even when the disease is confined to the joint itself, or its immediate neighborhood, Syme's or Pirogoff's operations are resorted to, and the healthy foot sacrificed.

So rare has been this operation at the ankle that Mr. Henry Hancock, senior surgeon to the Charing Cross Hospital, in an article in Braithwaite's Retrospect for January, 1860, states, "the operation was first performed by Moran, and subsequently by Jager and others abroad; but I believe that I am justified in stating that with the exception of those which I have done myself, there is not a single instance upon record in which excision of the ankle-joint has been performed in this country for disease."

In the English journals, I have not found other cases than those reported by Mr. Hancock, and in our own medical literature there is the same absence of cases. Why the solitary exception should be made of the ankle-joint—and the healthy foot sacrificed, I am at a loss to understand.

In both Syme's and Pirogoff's operations, in addition to the loss of the foot, there is the danger of sloughing of the flaps, or of bagging of the pus, which danger does not exist in resection of the joint.

In my own practice, an opportunity offered for an operation of this kind, though in a patient constitutionally unfavorable, and the result has been so satisfactory to the patient and surgeon, that it may not be devoid of interest to the profession. On the 26th of January, 1862, I was called to see Mr. J. C., a merchant about 45 years of age, who had received a severe injury by falling on the ice. On my arrival, I obtained the following history of the case: He was walking on the sidewalk, when he stepped on a spot of glare ice, which was concealed by a slight fall of snow, his foot slipped from under him, and he fell twisting it outward. Endeavoring to rise, he again slipped and wrenched the foot more violently than before. I saw Mr. C. about one-half an hour after the receipt of the injury. The foot was dislocated strongly outwards—the gastrocnemius muscle was exceedingly tense, reflex action being already aroused. On examination, the malleolus internus was found to be fractured, and also the fibula, about three inches from its lower extremity, the astragalus was rotated laterally so as to bring the outer articulating surface of the tibia to rise on the inner surface of the astragalus.

Reduction of the dislocation was found to be a work of some difficulty from the powerful tension of the muscles of the calf of the leg, now aroused to violent spasmodic action. The leg was flexed to right angles with the thigh to relax these muscles as far as possible, and with gentle, steady traction, the foot was drawn down and reduction accomplshed.

The reflex action was so strong that it was deemed best to keep the limb flexed on the thigh to relieve the tension of the extensor muscles; accordingly the limb was placed upon the double inclined plane, with the coaptation side splints and pads evenly arranged so as to give firm support to the side of the foot, and to obviate the danger of re-dislocation. The limb hardly had been thus dressed when it was re-dislocated with great violence, completely overturning the splint.

This reflex action becoming so violent, it was determined to place the patient in a fracture box, with the leg completely padded with bran in every direction, covering over the top of the leg for a couple of inches, so that the limb could not be thrown out of place by any violent action. This was absolutely necessary, as the sharp and jagged edge of the tibia, when the internal maleolus was broken off, was crowding firmly against the distended integuments, threatening to lay open the joint.

The fracture box was then swung so that whatever motion the patient might take, the limb would rotate without displacement. As the patient had been accustomed to the free use of alcoholic stimuli, a good allowance of whisky, with a strong anodyne, was given, but the patient had an uncomfortable night. The next morning the limb was swollen, with much discoloration; his nervous system began to give evidences of participating in the local trouble; there was a tremulousness about his hands, his tongue was covered with a yellowish white fur and tremulous, his pulse was irritable and rapid. Although anodynes and stimulants were largely used, they produced but little satisfactory results. The naps that he obtained were not refreshing or perfect, and he would start from apparent sleep with a shriek that could be heard for some distance, and when aroused he would complain of most intense pain in the limb. As it was evident that the patient would have a severe attack of mania-a-potu, and that the injury was a source of intense pain to him, partially kept under control by anodynes, counsel was requested, and I had the pleasure of receiving the advice of my instructor and friend, Dr. James R. Wood.

On the doctor's arrival, and my description of the injuries, he desired to examine the injury for himself. My experience of the previous night had not made me anxious to again attempt a reduction of the limb, and I did not urge an examination. Upon removing the bran from the top of the leg, the limb began to twitch spasmodically and the patient to shriek with the intense pain. The side of the fracture box was hardly loosened, before the limb was thrown out with great violence, and puncture of the integuments from the projecting jagged edge of the tibia seemed inevitable. The steady efforts of four strong men, under the able direction of Dr. Wood, were unable to reduce the dislocation. Chloroform was administered and the limb reduced; when the effects of the chloroform passed off, the dislocation again occurred, notwithstanding the endeavors of Dr. W. and myself to prevent it. The only way of obviating this violent action of the extensors, was to divide the tendo Achillis, which Dr. W. proposed, and it was accordingly performed. The limb was then replaced in the fracture box, and water dressings applied. Dr. Wood remarked that he had never witnessed so powerful reflex action. Notwithstanding the division of the tendo Achillis, the patient continued to complain of intense pain in the joint, returning in paroxysms of increasing severity.

Violent delirium followed, which the prodigal use of opiates could not control. The next morning vesications on the inner aspect of the limb showed that sloughing would ensue, and the patient was stimulated freely and the limb dressed with yeast poultices, to limit the gangrene as much as possible. The joint was, however, laid open by the progressive process of the disease, violent erysipelatous action accompanying it, running up the leg and side of the body; sympathetic buboes formed in the groin, and it seemed probable that the patient would succumb to the violence of the constitutional disturbance. As soon as the joint was laid open by the progress of the sloughing, a careful exploration was made with the finger, which revealed the hidden source of irritation. Within the joint were found several sharp spiculæ of bone, which had created the intense irritation, producing the violent reflex action which had become so early and so powerfully marked. When a limb is allowed to remain for some time with the dislocation unreduced, and the parts become irritated by the non-adjustment, it is not at all uncommon to have reflex action excited, which is rendered difficult to control; but in this case, where the cause of the injury was so slight, and the dislocation had been reduced so soon, the violent reflex action was a source of considerable mystery.

As irritation within the joint was removed, the intense spasmodic pain became relieved; the inflammatory action had, however, involved the periosteum, and large abscesses formed on the anterior and lateral aspects of the tibia—opening these the bone was found necrosed.

As the inflammatory action subsided, the prospects of saving the limb seemed so dubious, that amputation was advised by the surgeons who saw the case in consultation. On the anterior portion of the tibia, even as high up as within three inches of the head of the tibia, necrosed bone could be felt, and the exhausting suppuration was reducing the patient's strength.

My strong preference in favor of exsecting the joint finally prevailed, backed as it was by the argument that amputation could be easily performed if the exsection was not satisfactory, but that we could not so easily replace the limb after amputation, if from any reason we should find it desirable.

The method of operating was extremely simple, and for the main feature of it I willingly acknowledge my indebtedness to Dr. Wood. The malleolus and lower extremity of the tibia had become carious. The opening from the slough was enlarged by a straight incision along the inside of the tibia. The tibia was examined till it was found sound.

A strong curved needle was then carried round, closely hugging the tibia, and out through the original opening. A thread was thus drawn around the tibia, to which a chain saw was attached, and by this means the bone was easily divided at the point previously determined upon.

The exsection was completed by dividing the ligaments left, after the slough; as there was no union of the fibula at the point of fracture and the lower fragment was healthy, it was not interfered with; the interosseous, as well as the anterior and posterior ligaments, having been torn by the previous violent reflex action, there was no difficulty in carrying the foot up so that the astragalus should be kept in coaptation with the lower end of the tibia. Neither the anterior nor posterior tibial arteries or nerves had been interfered with in the operation, so that proper nutrition of the foot was provided for. The astragalus was examined and found not to be diseased; where the cartilage was involved it was removed, otherwise it was not interfered with. In the feeble condition of the patient, it was not considered judicious to keep him longer under the influence of chloroform; accordingly the gouging off of the necrossed portions of the tibia, higher up, was postponed. The limb was replaced in the fracture box and water-dresings applied. Profuse suppurration followed, but it was no longer of the intensely fetid character of the previous discharge. The patient's improvement in general health was marked; the drain of laudable pus from the system did not produce the constitutional disturbance that the previous unhealthy discharge had. The patient progressed most favorably, with no features of unusual character. Slight portions of bone exfoliated at the points where the old abscesses had formed. About the 1st of June, the union was sufficiently firm to allow the patient to move on crutches. He can now walk with only a slight halt, and the limb is far more serviceable than any artificial one could be.

The case was an unfavorable one for operation, from the previous habits of the patient, and from the prostration of the system by the violent constitutional disturbance produced by the spiculæ of bone within the joint, producing caries. The result is more satisfactory than any other operation that could have been performed, and I place it upon record, in the hopes that, at the present time, when injuries of articulations are so common, surgeons of eminence may turn their attention to this neglected field, and the saving of a part so important as the foot may be accomplished, where it is now sacrificed.

Accompanying the case the Doctor presented two water-colored paintings, which gave an accurrate view of the portion of the tibia removed; also a daguerrotype which gave an extremely faithful view of the limb when the patient first went out.—It did not give a fair view of the limb in its present state, as the muscles of the leg were wasted by want of use, and the provisional callus makes the ankle unusually large. The size of the calf of the leg is restored now by use, and the ankle much reduced in size by the provisional callus.

---

### QUARTERLY MEETING, JANUARY, 1863.

### *Aneurism of the Aorta.*

Dr. Enos presented a specimen taken from a man 32 years of age.

About the middle of December the patient came to his office with the aspect of a confirmed invalid. He complained of pain in the lower portion of the thorax, extending down to the epigastric region. It had existed for about six months, and had recently, become so severe as to render him unable to pursue his avocation of an engineer. His complaint, though severe, seemed to centre in his stomach, giving his disease the aspect of being a severe form of dyspepsia. He was, therefore, prescribed for accordingly. On the 29th of December the patient called again, complaining of increased pain in the epigastric region, accompanied by nausea, so much so as to keep him awake at night. So severe were the symptoms, that the doctor suspected spinal difficulty, although there was nothing in the symptoms to justify the conclusion. Anodynes were administered for the relief of pain, and the remedies, before described, continued. On the 31st of December a person called at the office for a certificate of death. About one o'clock of that morning the patient aroused his family, suffering extremely; he rapidly grew worse until he died, within an hour afterwards. Reflecting on his previous condition, in connection with the sudden and unlooked-for termination of the case, the doctor was led to suspect aneurism as the cause of death, and, on a post-mortem examination, such was found to be the case. On opening the chest, the right side was found to contain a large quantity of blood. To the right, and over the eleventh and twelfth dorsal vertebræ, lay a ruptured aneurismal sac of the aorta, adherent to the right lung above and closely bound down by strong fibrinous deposits to the spinal column, the vertebræ in immediate contiguity being abraded. The sac contained two openings, and it was difficult to discover which had ruptured, though the probability is that it was the one in contact with the vertebræ, as that part only contained the inner coat of the artery. No coagulae existed. The doctor accounted for the dyspeptic symptoms in this case by the pressure of the tumor on the splanchnic nerves, extending to the gastric branches. In relation to the symptoms, the doctor added, that Bennet had recorded a case of deep-seated thoracic aneurism that had been treated as chronic pleurisy.

Drs. Mason, Hart and Bell referred to similar cases, with symptoms more or less aggravated, simulating neuralgia, dyspepsia and asthma, without any evidence of aneurism until the post-mortem revealed it.

---

### REGULAR MEETING, JANUARY, 1863.

### *Rupture of the Ilium.*

Dr. Ball presented a specimen of Rupture of the Ilium, taken from a woman, who had died about twenty hours after having been knocked down and stamped upon by her husband. The post-mortem examination was made twenty-six hours after death, and besides the ruptured Ilium, there were several severe contusions on various parts of the body, particularly of the scalp. In the abdomen there was extensive inflammatory action, agglutinating the organs, and several patches of lymph.—The specimen suggested a *medico-legal* question of much interest and importance: "Whether the rupture was caused *by direct violence,* or by *inflammatory action?*"

Dr. Teller considered the time, after injury, too short to produce inflammatory action to such a degree, as to cause the rupture. He regarded it, therefore, as the direct result of violence.

Dr. Enos coincided with Dr. Teller, and cited a similar case, which proved fatal in fifteen hours.

Dr. Wm. Gilfillan cited two cases, one similar in its causes to Dr. Ball's, and the other produced by a blow from a bucket, which proved fatal within twenty-four hours.

Dr. Burge referred to a case where a man, pulling a rope, slipped his hold, and struck his elbow so violently against the abdomen, as to cause rupture of the intestines, that proved fatal in twenty-four hours.

Dr. Ball further enquired, whether there was in the cases cited, as in his case, much inflammatory action—and was answered by the gentlemen respectively, that such was the case.

### *Diptheria.*

Dr. Enos presented the *trachea* and primary branches of the *bronchi*, lined with *dyptheritic* membrane. The specimen was taken from a recently married woman, aged 21. She was taken sick on the 7th of January, but was not visited until the 11th of the same month. (She had never been sick before). Found her suffering from dyspnœa; the pulse was 120, the skin hot, and face flushed. Auscultation was indefinite, on account of various loud, sonorous and soft râles. Behind the left pillar of the soft palate was a diptheritic patch, while the faucus were red, and presented the appearance of having, recently, been denuded of diptheritic deposite. She was immediately put upon the tinct. ferri and brandy. Upon calling the next evening found her in a sitting posture, breathing loudly and laboriously—the face, neck and upper part of the thorax were swollen and crepitant, and visibly increasing. On puncturing the skin air escaped, but the part immediately refilled. She was painfully sensible of approaching dissolution. She died on the afternoon of Jan. 12th.

On post-mortem examination the *lungs* were found to be emphysematous throughout, interlobular and subserous; and the cellular tissue of the *anterior mediastinum* inflated. The *larynx* was eroded, and above the *ventricles* there was a pendulous mass of the size of a pea, that had evidently, been an obstacle to respiration. The *trachea* and *bronchi* were livid with a continuous tube of diptheritic membrane to the extent of the cartilages, while some of the smaller ramifications were filled with dense threads of diptheritic matter. On inflating the lungs under water, the air cells were found to have given way in consequence of the rupture of the smaller bronchial tubes, and the air had thus found its way into the parenchyma of the lungs, and become interlobular and subserous; thence the air traversed the cellular tissue, beneath the pleura pulmonalis, and along the root of the lungs into the anterior mediastinum to the lateral boundary of the pleura, and through the thymic opening of the thoracic fascia into the areolar tissue of the neck.

*Perforation of the Stomach.*

Dr. John Hill showed a specimen of perforation of the stomach, accompanied by the following report of the case:

H. M. I., a law student, aged 24, of Schoharie county, N. Y., had for several years been a dyspeptic; was much emaciated, sallow, and had suffered from frequent billious attacks. For some four weeks previous to his death, he had been on a visit to his friends in this city. About 5 o'clock, P. M., December 30th, he returned from a visit to New York, and was subsequently engaged playing the flute for some time, apparently as well as usual. At 7 o'clock he set out to return to New York, but on reaching the ferry, was suddenly seized with a pain in his bowels, so severe as to occasion his return. The doctor was first called to see him soon after. About 8 o'clock. P. M., found him sitting up, suffering from an intermitting pain in abdomen, at times very severe. His pulse was slightly accelerated. Tinct. opii was administered twice to relieve the pain, and subsequently a mustard plaster was applied over the region of the stomach to counteract vomiting which ensued. The matters vomited were principally liquid, of a dark green color and very acid. After vomiting several times, he expressed himself as feeling much better, Seven pilula cathartica comp. were ordered, four to be taken as soon as the stomach was sufficiently settled to retain them, and the other three to be taken in the morning in case bowels were not open. Two or three hours after taking the first dose of pills, he vomited, and once subsequently during the night. On the following morning, there having been no passage from his bowels, the other three pills were taken according to directions.

Five o'clock, P. M., December 4th, found him again sitting up. He conversed freely in relation to his illness, stated that he had severe pain early in the morning, and had taken more laudanum, had suffered comparatively little during the day; nothing had passed his bowels, nor had there been any more vomiting; had been up most of the day. He complained of a sense of fullness in his abdomen, and of some little difficulty in voiding his urine. The tongue was covered with a thin white coat, and the pulse was tense and frequent. Directed fomentations of hops to the abdomen, and an enema, to be followed by a purge, if necessary.

Called again at 11 o'clock, six hours after the last prescriptions, and found him dying. He died at 12 o'clock.

*Post-mortem* examination five hours after death. Drs. Teller, Marvin, Henry, Hutchison, Griggs and Wilson were present. Upon laying opon the abdomen, found extensive peritoneal inflammation, fibrinous exudations, agglutination of the organs, and about a pint of serum. Tracing the stomach near the piloric extremity, its coats were thickened and adherent posteriorly to the contiguous parts. Upon the anterior face of the stomach, two and a half inches from the pilorus, there was an oval-shaped opening, three-eighths of an inch in its longest diameter, from which issued a green colored liquid- The stomach, on being laid open on the line of its lesser curvature, presented the following appearances: The greater portion of the lining membrane was of a lead colored hue, and as it approached the perforation, was of a dull white or reddish cast; the thickened coats of a doughy feel, some. what resembling tripe, and all that portion immediately around the perforation, and onwards through the pilorus and in the upper third of the duodenum, was in a state of inflammation. The inner orifice of the perforation was found to be considerably larger than the outer, the coats of the stomach thickened to some three-eighths of an inch, the edge of the perforation and the membrane around the internal orifice jutting out on one side so as to overlap the passage, produced probably by the loose, unresisting areolar tissue being pushed forward by the swollen and thickened condition of the deeper tissues at that point.

That the hole in question was formed by the process of ulceration there can be no doubt, the ulcer being located upon the mucus membrane and traveling outwards, extending in circumference and depth, gradually sweeping away the substance of the areolar and muscular tissues until the peritoneal membrane was reached, which, owing to deficient nutrition in consequence of the destruction of the subjacent tissues, gradually perished. The slough for a time retaining its position with a feeble hold upon the living parts, when, either by distension of the stomach or some violent effort, it suddenly gave way and the gastric fluids passed into the abdominal cavity, thereby causing peritoneal inflammation, without exhibiting the usual symptoms, and passing through the various stages of peritonitis to a fatal ter mination within twenty-eight hours from its first manifestation.

Dr. HUTCHISON regarded Dr. Hill's case as an exceedingly interesting one in all respects. He was especially struck with the valve-like opening through the walls of the stomach, seeming to indicate that the opening may have existed for a considerable period of time before death, though such was not probably the case. According to one other case which had fallen under his own observation, and according to the literature of the subject this case is in many respects typical, showing by the form of the ulcer, as well as by the insidiousness of the symptoms, that it was of long standing. The disease most frequently occurs in persons of sedentary habits and of enfeebled constitutions, such as seamstresses and milliners, and usually, as in this case, there is but a single ulcer. In rare instances, however, there are several.

### *Deformities of the Feet and their treatment by means of Plaster of Paris.*

By Dr. D. C. ENOS.

I present this cast of a severe form of club foot (varo equinus) in order to bring before the society the important subject of deformities of the feet, and more especially to explain a simple mode of managing them.

Every one who has had much to do with these unfortunate cases, is aware of the difficulties attending their adjustment and cure. It not unfrequently happens that inflammation, abrasion, ulceration, abscess, or irritative fever, is caused by the mechanical appliances made use of to correct the deformity, necessitating at least a temporary suspension of the treatment. This is unfortunate, not only on account of the pain produced, but on account of the actual retardation of the cure over and above the loss of time by the cessation of the treatment; for the inflammation frequently increases the quantity of adventitious matter to be absorbed, and for a long time the parts are too tender to bear the same amount of corrective force they would before. There are other reasons connected with the divided tendons which we shall explain hereafter.

The patient, or the paitent's friends, sometimes become discouraged, and not inaptly call the process *torture*, and withdraw the case from the surgeon, declaring they prefer the deformity to its removal at such a cost, But even when the surgeon has the opportunity to continue the treatment of the worst forms of talipes, one that has for five years been used in walking, he may, after all, fail to restore the foot to its normal position.

Again the mechanical appliances are more or less complicated, requiring frequent alterationss and new contrivances to adapt them to the various cases, and to different stages of the same case, being therefore, very inconvenient for the surgeon, as well as expensive to the patient.

A mode of treatment, therefore, which is cheap, simple, everywhere accessible, readily applied and adapted to all cases and conditions; one which does not irritate or inflame, and hence can be more encouragingly and efficiently, because *continuously* applied; one, in short, which is painless, speedy and certain in bringing the foot to its normal position, is a desideratum to the surgeon, and a boon to humanity.

Such I find, in the management of these difficult cases, to be the actual working of appliances made of plaster of Paris and strong muslin.

The mode of using it is as follows: Take a straight piece of muslin, wide enough to embrace or nearly embrace the head of the tibia, and long enough to extend down from the head of the tibia around the heel and as far as the great toe; then *tear* five or six other pieces of the same size; next stir some plaster of Paris in a little warm water till it is about the consistency of cream; place upon a board or table one of the pieces of muslin, and put on it a teaspoonful or two of the plaster, and with a long knife spread it evenly over the cloth, so that it shall be *wet* and *thinly covered*; place upon this another piece of muslin smoothly, and with more of the plaster spread in like manner, and so continue to spread layer after layer until the last, which should *not* be spread, or if so, should be covered with a thin layer of raw cotton, to come next the limb. Place the bandage behind the leg and first bend it around the head of the tibia and let an assistant hold this firmly, while you next apply it to the sole of the foot as far as the great toe; if it should be too long, turn it back on itself, so as to leave the toes exposed; next bend it around so as to neatly embrace the foot to the instep. There is now a redundancy of cloth at the angle of the foot, which may be disposed of by folding it on itself, so as to smoothly apply the remainder to the ankle and leg. Over this a roller should be closely applied from the toes to the knee and back again, so that the dry cloth may absorb the moisture from the plaster, and thus facilitate its setting. As soon as the roller is applied, grasp the foot and ankle firmly, and forcibly press the foot towards its normal position, holding it steadily ten or fifteen minutes till the setting has made it firm.

Sometimes I embrace the foot and ankle first, by taking a piece of muslin from one to two yards long, and from six to eight inches wide. Spread it thinly with the plaster, double it longitudinally and spread it again and double it once more, and apply it to the foot and ankle somewhat like an ordinary roller, holding the foot as before till the plaster sets. This answers for deformities of the foot, simply, where its position with the tibia is normal; but if this is abnormal, as it usually is, the retentive apparatus must extend to the knee, after embracing the foot, as already fixed. Sometimes this can be advantageously done by putting a number of folds of the plastered muslin along on the top of the foot and anterior part of the leg, strengthening it in the instep by an extra quantity of plastered cloth, binding this on with a roller, and holding the foot firmly up, till the plaster sets. Occasionally, to strengthen the first described application, I apply over it this plastered and four folded roller around the foot and ankle, thus making a figure of eight bandage.

After the apparatus has been on for three or four days, it can be removed and a new one applied, when from the absorption that has taken place, an increased amount of pressure can be borne and the foot be made to approach still more towards its normal position.

I have now four cases under treatment, but the detail of the following one will suffice as an example of the treatment pursued:

C. H., aged 6, a resident of the central part of the State. Sometime in the dental period of the first year, the mother says, his left leg and foot became paralyzed, there being both loss of motion and sensation. Soon, however, motion was restored (if ever lost) in the flexor and abductor muscles, as was evinced by the deformity which began to appear.

The triceps and posterior tibial, the short flexor of the foot, the abductor pollicis became so much contracted as to draw the foot up in the position you see, viz., the heel drawn up by the gastrocnemius soleus and plantaris; the tibial muscles have drawn the inner margin of the foot up so that the tuberosity of the scaphoid touches the internal malleolus, while the short flexor of the toes and the abductor pollicis have so bent the foot on itself that the toes approach the heel, and the foot points toward the opposite ankle. Nothing was done to rectify the deformity. He has walked with it in this position for five years, the weight of the body, when sustained by this foot, resting upon the *dorsal aspect* of the bones of the tarsus, while at this place had accumulated a large mass of elastic adventitious substance which the photograph shows better than the cast. The foot has frequently been burned, sensation being lost, and once his mother drew a needle from it which had, some time previously, been run in its entire length. The limb is somewhat attenuated, as is usual in cases of talipes.

Sensation is now restored, but the muscles, supplied by the external popliteal nerve, are still nearly motionless. Electricity has been used.

Four weeks since, in the presence of Drs. Conkling, Spier and Ormiston, while the boy was under the influence of chloroform, I divided subcutaneously the tendo Achillis, the planter aponenrosis and short flexor. The tibial muscles and the abductor pollicis were not divided. Small pieces of adhesive plaster were placed on the punctures through the skin and compressed, held on for a time to prevent the effusion of blood into the tissues and sheath of the tendons. The plaster dressing was then applied in the manner already described, and while it was soft the foot was somewhat forcibly and steadily held as near the normal position as possible. When the plaster became hard and dry, the boy was put to bed. The next day I found him cheerful, and amusing himself with his playthings. The foot had not been in the slightest degree uncomfortable. On the fourth day I removed the plaster; it was hard in all places except that covering the heel. This he had broken by striking his heel on the floor as he was playing about the room in a sitting posture. The pressure has been so uniform that there was not the slightest sign of excoriation or a single tender point. The limb was washed and rubbed, and considerable passive motion made use of. A very perceptible improvement had taken place in the position and contour of the foot. The adventition substance on the dorsal aspect of the tarsal bones was noticeably diminished. The apparatus was reapplied as before, and the foot again forcibly and steadily held towards the normal position, pressing the ball of the great toe outward, and the fifth metatarsal bone upward, the whole foot flexed as much as possible upon the tibia. It is now a month since the operation. I have applied the apparatus about twice a week, making passive motion at each dressing. It has not given him a moment's pain or uneasiness, no abrasion, and the treatment has been continuous. To-day the sole of the foot is readily placed at right angles with the tibia, and he voluntarily, when the dressing is off, places it properly upon the floor.

The adventitious substance is nearly all gone, and the foot has assumed quite a normal appearance. The tendo Achillis has united. An ordinary shoe will now fit the foot, which must be kept in position by the usual mechanical contrivance, until the paralyzed muscles are restored, or the deformity will reappear.

In the other cases the apparatus has been quite as satisfactory. One, a case of double varo-equinus, was an infant operated on when a month old. The tendo Achillis was divided. The apparatus gave no inconvenience, the child only crying when the dressing was applied. The child's father had double club foot. There were ten children in his father's family, and six of them had club feet. He, himself, was operated on many years ago by Dr. Detmold, who has the honor of first introducing orthopedic surgery into this country. No doubt he was treated with all the skill of that distinguished surgeon, yet he suffered greatly; abscesses formed about the foot which were troublesome to heal, requiring a long intermission in the mechanical treatment, a re-division of the tendons, and finally a failure to bring the foot fully into the normal position. The inner margin of the foot is too crescentic, and the weight of the body in walking rests painfully upon the fifth metatarsal bone. Still he was greatly benefited by the operation. As he observes, therefore, the steady, uniform, painless and successful working of the apparatus upon his child, he frequently and feelingly comments upon its advantages. On this infant I sometimes allow the application to be on five or six weeks without changing.

Morbid Anatomy.—Scarpa says, that the appearances presented on dissection, are not necessarily identical, even in the same form of distortion. But Dr. Little justly remarks, that it is in the infantile and unused foot that we must look for the true anatomy of these altered relations, and not in feet whose deformity has been increased by locomotion, or by improper mechanical means in treatment. Sir Charles Bell said he found, on dissection, less defect than could have been imagined. Nelaton asserts, at first the bones of the club feet in infants are not notably deformed. At a later period of life they are atrophied in some and hypertrophied in others, and hence marked changes in their configuration. He thinks, therefore, children should be treated at once, as there is a consecutive deformation of the bones and retraction of the muscles. Traction in such young children, he remarks, suffices without an operation.

"A contracted state and atrophy of the muscles, are not incompatible with each other. There is a kind of paralytic loss of power in muscles, which is combined with contraction. We have seen the gastroanemii both contracted and atrophed at the same time."—(Muller.)

In the 3d volume of the Transactions of the London Pathological Society, Mr. Adams described the morbid anatomy of two cases of dissected club foot. One patient had one equino-varus, and the other one equino-varus, and one taliepes-varus. The bones in the equino-varus were altered, partial luxation of the astragalus forwards and downwards, produced by the almost vertical position of the os calis—partial luxation of the navicular bone inwards, so that its internal border closely approaches the inner malleolus, the external half of the head of the astragalus being exposed.

The cuboid bone was also partially displaced inwards from its articulation with the os calcis, following, but not to a proportionate extent, the navicular bone.

The anticular cartilage of the exposed parts of the astragalus was removed, and the bones atrophied. The bones in his cases were distorted and altered in shape, according to the different muscles brought to bear on them and the surfaces exposed.

The muscles were generally found atrophied and fatty. Mr. Broadhurst, vol. 10, Trans. London Path. Soc., gives an interesting dissection of a non-congenital club foot of fifty years standing. The bones were found changed and fragile, and the muscles more or less degenerated. In most cases the bones are atrophied and soft, but occasionally they are hypertrophied, and those parts bearing unusual pressure are eburnated.

Some ligaments are shortened, others are elongated and occasionally some are absent.

### *The Causes of Club Foot.*

On this interesting subject, authorities are not agreed, or at least have not been. Glisson, Camper and Blumenbach advocate the opinion that the primitive formation of the bones is incomplete.

Scarpa, in his memoir, remarked that the deformity of the individual bones was slight, and that the relation of the astragalus to the anticular surfaces of the tibia and fibula was comparatively but little disturbed. Dr. Little's dissections have led him to confirm this opinion of Scarpa; Jorg, Colles and Cruveilhier attribute the deformity in most instances to malformation of the bones, which they consider a serious, if not insuperable obstacle to the reduction of the foot to its normal position. Delpech, in 1823, held this opinion, but he subsequently subscribed to the hypothesis that the deformity is produced by the contraction of some muscles and the relaxation of others, the bones being originally well formed; or in other words, that the deformity is caused by abnormal innervation. Rudolphi first directed attention to the nervous centres as the possible seat of the primary disturbances in most instances. Violent, convulsive motions of the fetus in utero, and the early period (three to five months) at which the deformity sometimes occurs, he refers to as proof of his opinion. In the museum at Berlin there are specimens exhibiting malformation or deficiency of the cerebrum and medulla spinallis, some anencephalus and some hemicephalus, both the hands and feet being affected in the same manner, showing the great influence of nervous centres.

Mr. Guerin says that "a great many observations made on monsters and on the normal fetus have proved that a relation exists between congenital muscular retraction and congenital alterations of the cerebro-spinal system. Mr. Adams asserts that talipes varies in both feet; usually, if not always, co-exists with spina bifida in the lumbo sacral region, from deviation or irritation of the cauda equina" This I am sure is not always the case, for I have seen spina bifida in this region in children otherwise normal. "When club foot exists together with other deformities of joints, they are all the result of convulsive retraction of the muscles characterized by an extreme shortening of the muscles and extremities." He rejects, therefore, very justly, the notion of Cruveilhier and others, that the deformity of the bones is primary, and is produced by compression of the uterus upon the fetus in cases where the liquor amnii is scanty. M. Duval also says "the most frequent cause of club foot is a lesion of the cerebro-spinal system Infants born with paralysis of one or both sides are frequently affected with club foot on the paralyzed side. The paralysis may disappear after a few months, and the deformity remain, or it may persist and the limb become atrophied. Eight out of ten cases of consecutive club foot arise from inflammation of the cerebro-spinal system, and are preceded by convulsions, paralysis or muscular contractions. Truly may not the same be the cause of the congenital disease?" Hereditary transmission, and hence the early alteration of the germ is a frequent cause of club foot.

This is an interesting subject to the physiologist, though not yet fully understood. Nelaton says that "the anatomical elements have the property of giving birth to elements like themselves, or to determine, in their neighborhood, the generation of elements of the same kind. That organic substances enjoy the property of transmitting, by simple contact with substances of another kind, the peculiar molecular condition that some exterior circumstances have produced in them." The fact is well ascertained, notwithstanding our inability to fully understand and explain it. I have alluded to one case I have now under treatment, of double equino-varus, whose father had the same congenital deformity—six out of ten children in the father's family had club foot. There can be no doubt that the germ is early impressed in utero with the imperfections of its parent. "There is," says Paget, "in all science, no fact so strong as this, and yet it only illustrates the common rule of the transmission of hereditary properties, whether natural or morbid."

The *healing of divided tendons* is a subject interesting both to the physiologist and the practical surgeon.

It has been investigated by many observers since the days of Hunter (1767), mainly by experiments and observations made on the lower animals. Recently (1859) Mr. William Adams has studied the reparative process in human tendons after the subcutaneous division for the cure of deformities. (See Medico Chirurg. Trans., vol. 42.) Without following these and the host of experiments that arose in the intervening period, (such as Mayo, 1827, Von Ammon, 1837, Pirogoff, 1840, Bonvier, 1838, Paget, 1849, Gerstaecker, 1851, Thierfelder, 1852, Boner, 1854, and others,) it will be sufficient for our present purpose to refer to some points which may be regarded as established, and which have a practical bearing on the subject. Tendons are now cut sub-cutaneously. The first operation on the tendo Achillis, by Lorenz of Frankfort (1789), was down on the tendon through the integument. The first effect of the division of the tendo Achillis is retraction and separation of the divided extremities, from about half an inch, in infants, to one or two inches in the adult, varying according to the c ntractility of the triceps and the flexibility of the ankle-joint. The cellular sheath of the tendon is not usually divided, as Mr. Paget thought, but being elastic and loose it yields before the tenotome, while the firm tendon is cut. A little blood is effused into the undivided sheath and sursounding tissues. This blood retards rather than facilitates the repair. Its more solid portion remains sometime, inclosed as a foreign substance in the new tendon. (Paget.)

Compression should therefore be made immediately after the withdrawal of the knife. A short time suffices to stop the oozing. The reparative material is a nucleated blastema thrown out from the divided tendon, but mainly from the cellular sheath, which maintains a tubular connection between the divided extremities, and this gives form to the new tendon.

The inflammatory process retards the healing, therefore too much force must not be used in attempts at reduction. The cut ends of the fibres of the old tendon swell and split, and the more delicate fibres of the new shoot in between, thus dove-tailing the two together. The new tendon is grayer, more translucent, and less pearly in appearance than the old. Its fibres are smaller and less separable. The new tendon is not absorbed, but remains through life. Hence time does not bring about a *linear cicatrix*, (i. e.) a contraction and final obliteration of the new tendon and a reunion of the old cut surfaces, as is maintained by Messrs. Tamplin, Brodhurst and Coote. Therefore, if deformities recur, contraction of the muscle and not of the new tendon, is the cause. The new tendon is more adherent to the surrounding tissues than the old, and never has the same freedom of motion. Hence secondary operations are of little avail, and the twentieth one of Dieffenbach, in one case, probably did but little good. Tendons inclosed in dense tubulor sheaths, and almost devoid of cellular investment, like that of the posterior tibial as it passes behind the internal malleolus, sometimes fail to unite, if the separation is more than a quarter of an inch, because there is no *cellular* sheath to pour out the reparative material. The cut ends become adherent to the dense tubular sheath, thus destroying or impairing the use of the muscle.

Tendons thus situated, therefore, should not be divided, and if the division be made *near* such tubular sheath, separating force should not be applied for four or five days, and then very gradually.

The division of the tendo Achillis is less objectionable than that of any other tendon, because of its complete areolar investment and isolation from dense structures, thus leaving considerable freedom of motion after union—at least after the first.

The division of the short flexor being always made near its origin from the os calcis, is obviously free from the objection. From the law then, which seems to govern the repair of tendons, we may, I think, infer that as a general rule for the cure of any given deformity of the foot, other things being equal, the fewer tendons cut the better. Hence, too, the questionable practice of those surgeons who resolutely divide everything that opposes the reduction of the foot to its normal position. Especially is this true in recent cases in which the limb is first affected with paralysis, either partial or complete, and the muscles are for some time, as M. Guerin has said, *retracted*, rather than actually shortened. It is easy, therefore, to see the utility of an apparatus that can, by its equable and well diffused points of pressure, gradually, and without pain or inconvenience to the patient, overcome the deformity in most cases of talipes, without recourse to tenotomy, or at least the division of those tendons whose future play would thereby be embarrassed.

*Is the mode of treatment of club foot with plaster of Paris new?*

Having used in fractures splints made of muslin and plaster of Paris with much satisfaction, I determined to try it in deformities of the feet. I found it to answer my expectations well. This mode af treating talipes was *new* to *me*. I was not aware that any one had either used or recommended it, and yet it seemed so simple, I wondered it had not been tried.

Since I commenced this paper for the society, I have examined as far as practicable the literature of this subject, to see if any reference is made to this mode of treatment. I find no allusion to it in the text of our standard works on surgery, nor in any recently published lectures on orthopedic surgery. I judged, therefore, that if this mode ever had been used, it had fallen into disuse and forgetfulness.

I find the reviewer of Sir C. Bell's work on surgery (British and For. Med. Rev. 1838,) says, "by the hand alone, in infants, we can change the direction of the parts without section, and readily enough maintain them in their new position by plaster of Paris." In 1836, Pauli said, two or three days after section, he surrounded the foot in a mould of plaster of Paris, which he made in a jointed wooden boot, and allowed it to harden. If the proper position was not at once effected, he renewed it frequently. (Bulletin de l'Academie de Medecine de Paris, 1836.)

Dieffenback, in 1839, said, though he generally used Stromeyer's or Scarpa's apparatus in extreme cases and irritable subjects, the limb was evenly bound with a roller from the toes to the calf, and the bandage then saturated with boiled starch or a solution of resin in alcohol, or with thin plaster of Paris. He then directed the patient to stand upright and press the bound-up foot firmly on the ground, and remain in that position till the bandage had become stiff and dry.

For a period then of nearly twenty-five years, I have found no one advocating or adopting the treatment of talipes with plaster, with the exception of an allusion to it by Nelaton in 1855. He put a plaster of Paris splint on the inner side of a case of varus, and when it was cold held it in position with strips of India rubber. This he said he "imagined at the time." Evidently then he was not aware that plaster had been suggested for such cases. These imperfect allusions to an important mode of treatment prevent our calling it *new*, though, like Nelaton, we "imagined it at the time."

If any one of those referred to had perfected a simple and effective mode of application, doubtless it would not have fallen into disuse and forgetfulness.

The profession were not aware of the utility of silver wire suture in vesico-vaginal fistula, till Dr. Sims perfected its application, notwithstanding it had been used by Bossuet many years before.

Now that the attention of the profession has been definitely called to the practical mode of treating deformities of the feet with plaster of Paris, such I think are the advantages secured by it, that the surgical "world will not willingly let it die."

